# Supplementary material for: Exploring the p53 connection of cervical cancer pathogenesis involving north-east Indian patients
Source: PLoS One. 2020 Sep 25;15(9):e0238500. doi: 10.1371/journal.pone.0238500 (PMC7518589; doi:10.1371/journal.pone.0238500)
Supplement: S1 Table — (DOCX) [file pone.0238500.s001.docx]

Supplementary Table: Details of p53 exon4 polymorphism based difference in genotype analysis

| **Stages** | **HPV +ve (N=72)** | | | | | **HPV -ve (N=13)** | | | | |
| --- | --- | --- | --- | --- | --- | --- | --- | --- | --- | --- |
|  | N | Pro/Pro | Arg/Pro or Arg/Arg | P value | ODDs ratio | N | Pro/Pro | Arg/Pro or Arg/Arg | P value | ODDs ratio |
| **IIA and IIB** | 48 | 9 [18.75] | 39 [81.25] | ref | 0.877 (0.258-2.979) | 09 | 5 [55.55] | 4 [44.45] | ref | 1.250 (0.118- 13.240 |
| **IIIA, IIIB**  **and IV** | 24 | 5 [20.83] | 19 [79.17] | 0.834 |  | 04 | 2 [50.00] | 2 [50.00] | 0.859 |  |
