# Supplementary material for: Exploring the p53 connection of cervical cancer pathogenesis involving north-east Indian patients
Source: PLoS One. 2020 Sep 25;15(9):e0238500. doi: 10.1371/journal.pone.0238500 (PMC7518589; doi:10.1371/journal.pone.0238500)
Supplement: S1 File — (DOCX) [file pone.0238500.s002.docx]

**Uncropped images and blots incorporated in the Manuscript**

**
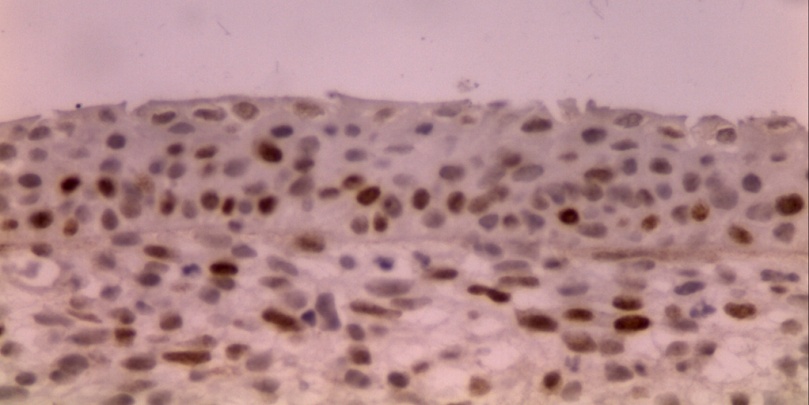
 Control**

**
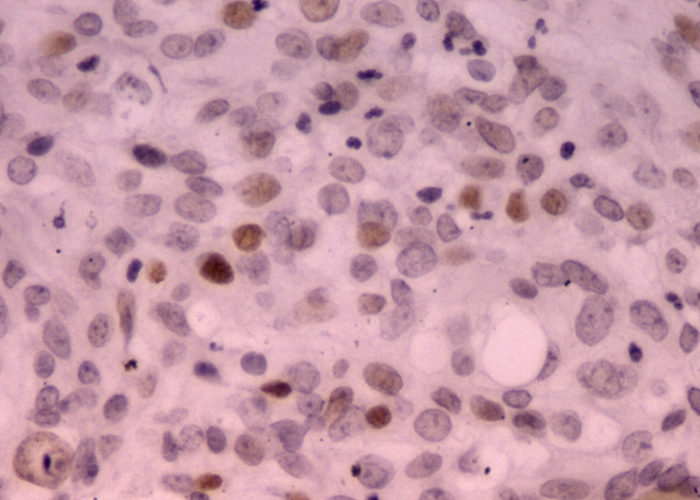
 CaCx IIB**

**
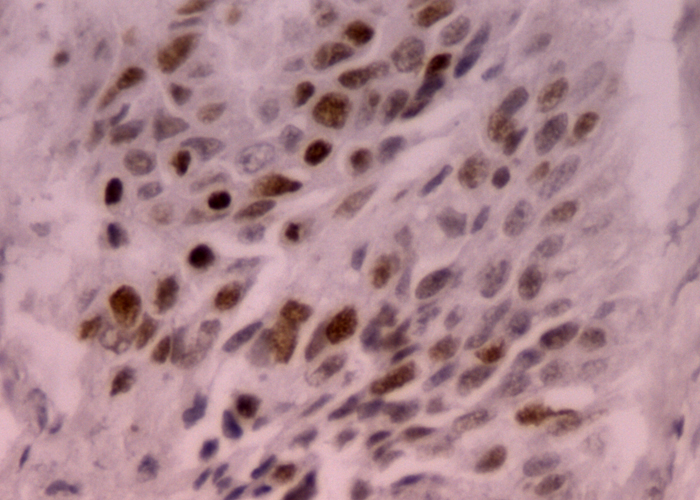
 CaCx IIIB**

**
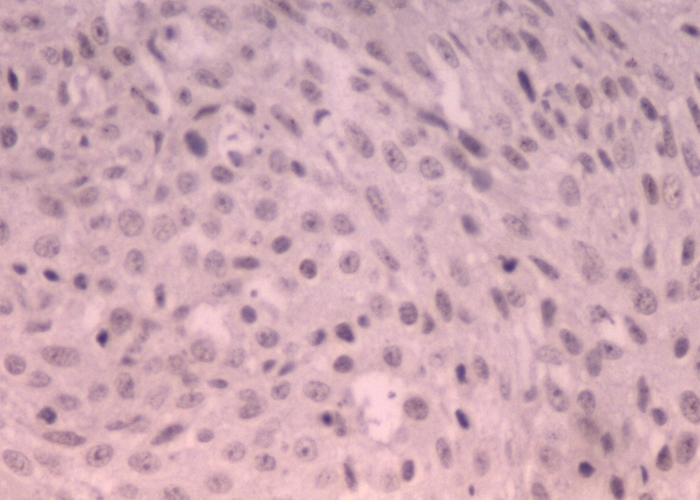
 Negative control**

**Fig 3: Representative panel of IHC results showing downregulation of p53 expression in HPV related cervical cancer cases especially in lower grade of CaCx cases (Stage II) compared to higher grade (Stage III).**

**Representative *p53* exon 4 sequencing**


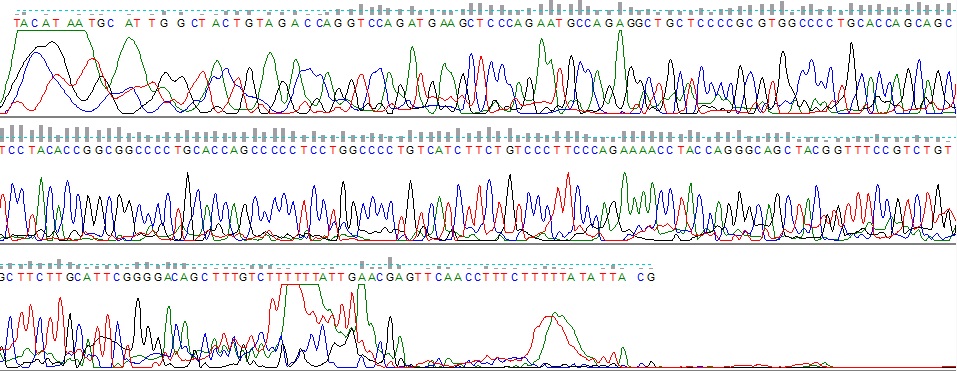


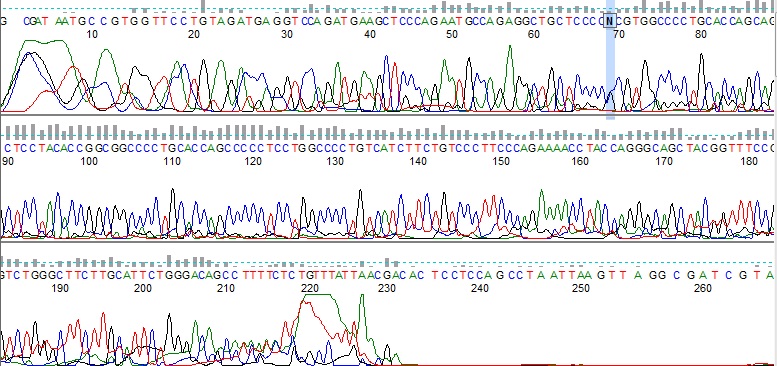


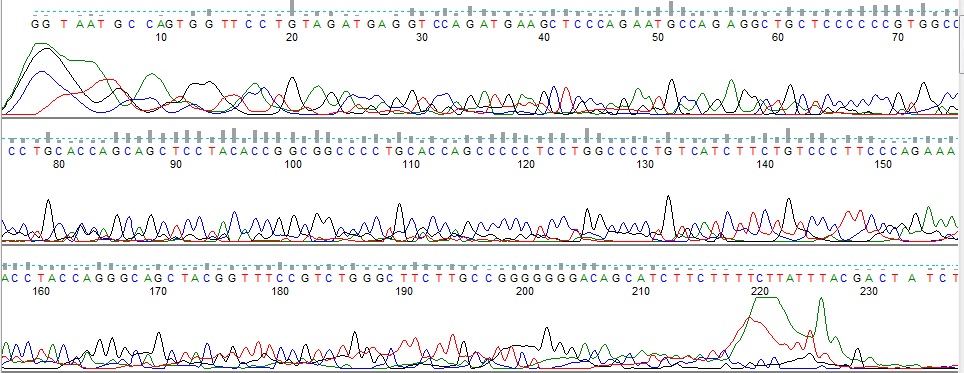


**Fig 4 B Representative electrophoregrams showing the presence of Arg/Arg, Arg/Pro heterozygous and Pro/Pro genotype based on p53 exon 4 sequencing analysis in the studied Cervical cancer patient population cohort**

**
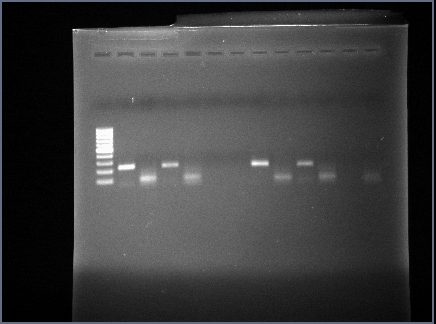
**

**Fig 5. Representative agarose gel electrophoresis photograph showing p53 methylation profile analysis by MSP methods in Cacx (Cervical Cancer) and Con (Non neoplastic Control) cases; M represents Methylated and UN represents Unmethylated based assay.**

**
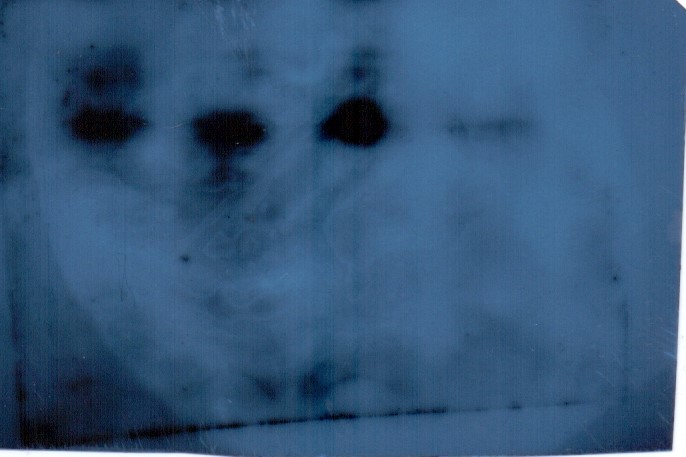
**

**
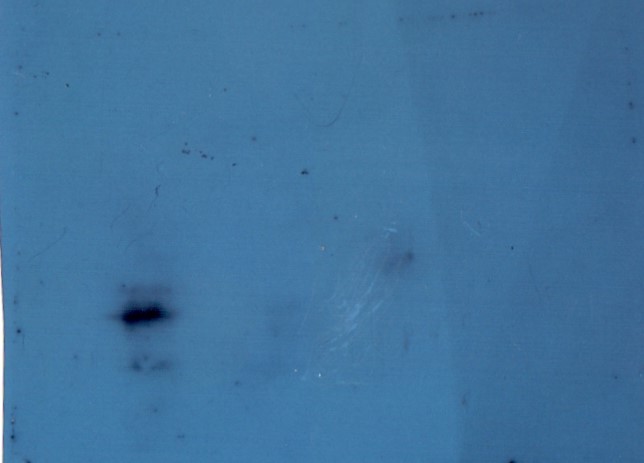
**

**
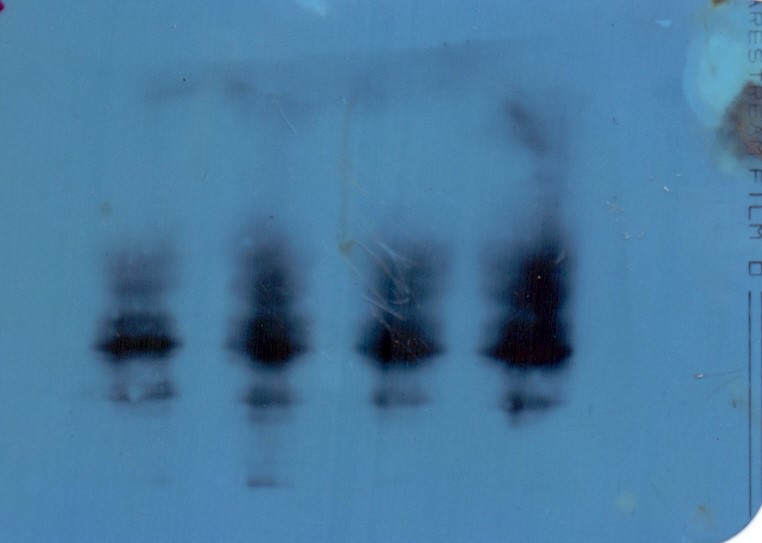
**

**Fig 6: (A) Representative western blot showing the absence of p53 acetylation in HPV positive cervical cancer cases compare to non-neoplastic control at site K305.**

**
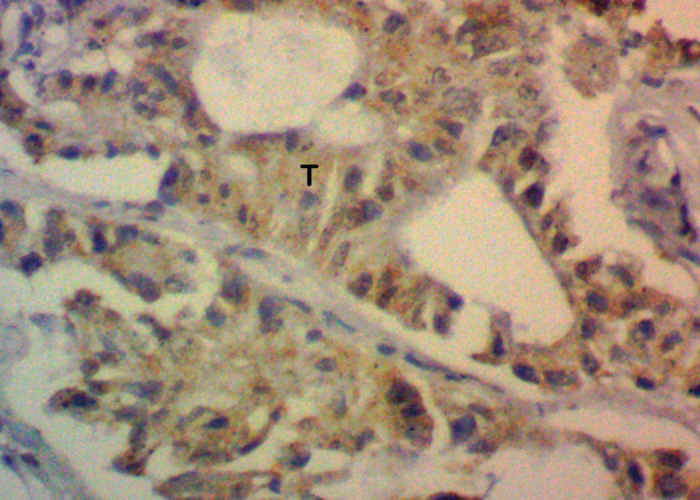
**

**P53 56A K-373 400X**

**
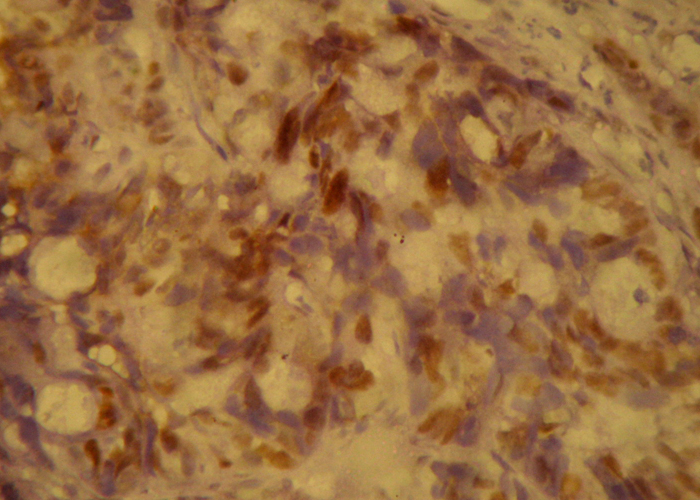
**

**P53 2607-14 K-373 400X**

**Fig 6 (B) Representative panel of IHC showing the presence of p53 acetylation in HPV positive cervical cancer at site K373. The positive results were observed in very few cases**

**
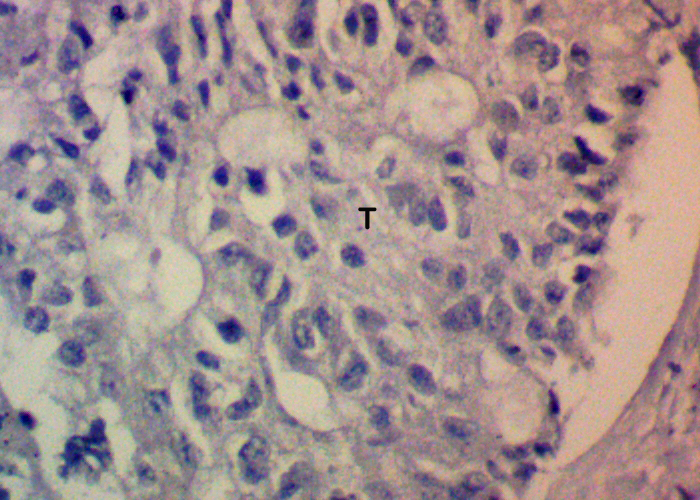
**

**P53 56A K-382 400X**

**
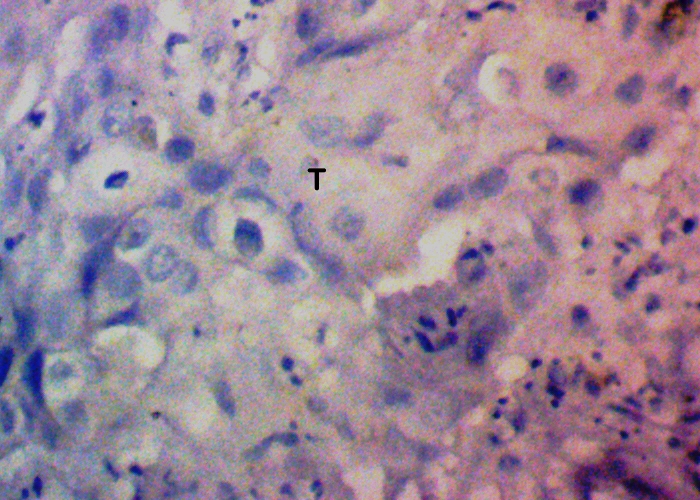
**

**P53 2607-14 K-382 400X**

**Fig 6(C) Representative panel of IHC showing the absence of p53 acetylation in HPV positive cervical cancer at site K382**
