# Supplementary material for: Exploring the p53 connection of cervical cancer pathogenesis involving north-east Indian patients
Source: PLoS One. 2020 Sep 25;15(9):e0238500. doi: 10.1371/journal.pone.0238500 (PMC7518589; doi:10.1371/journal.pone.0238500)

SciGenom Trace Viewer

Sample :E4.4_E4.FP_26280-1_8173
Trim Start :83
Trim End :107
Qv20 Bases :24

Run start: 2016/02/23 19:11:33
Run stop: 2016/02/23 21:23:31
PDF created: 2016/02/24 09:05:30

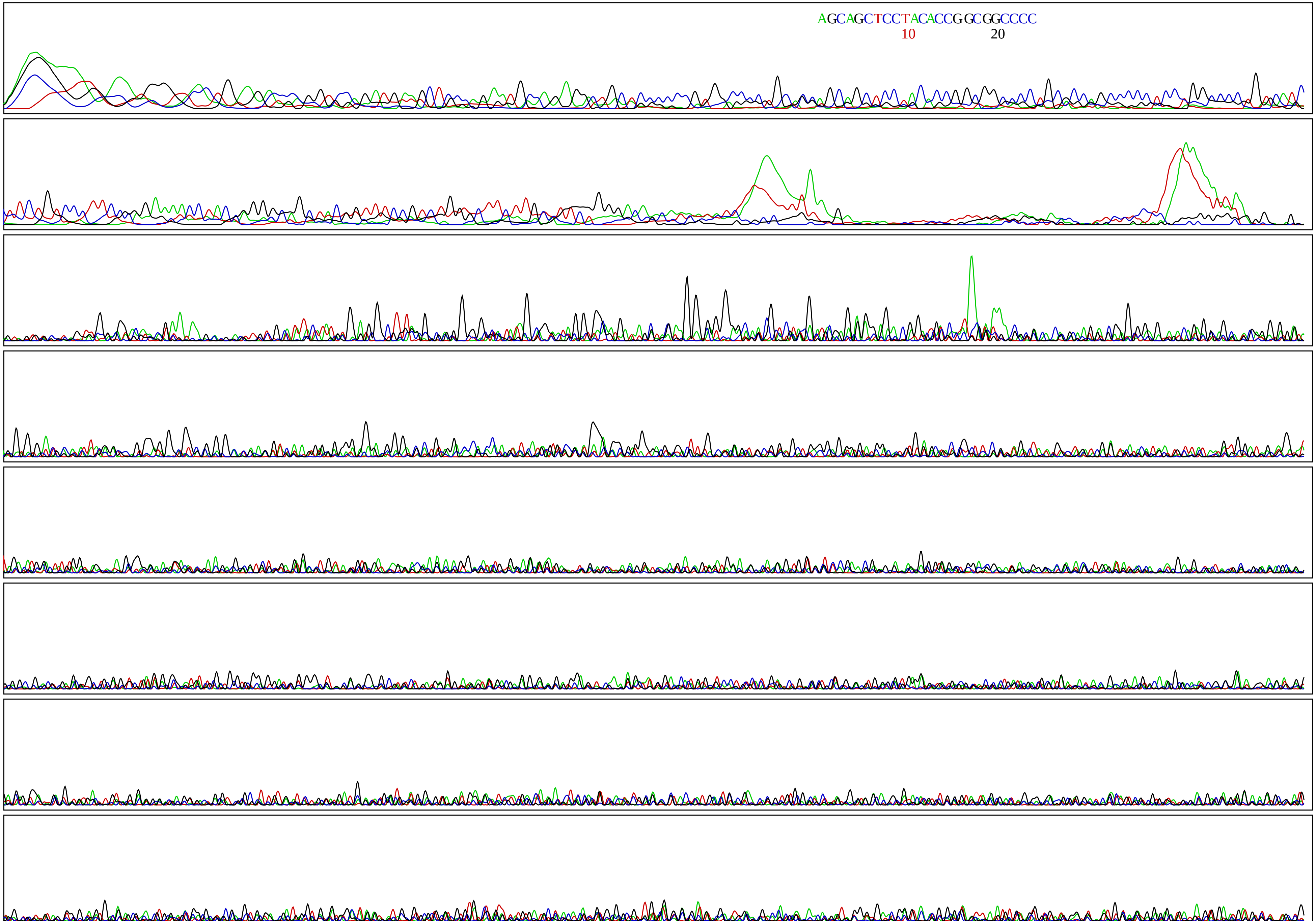

Supplement: S3 File — (PDF) [file pone.0238500.s004.pdf]
