# Supplementary figures and images for: Exploring the p53 connection of cervical cancer pathogenesis involving north-east Indian patients
Source: PLoS One. 2020 Sep 25;15(9):e0238500. doi: 10.1371/journal.pone.0238500 (PMC7518589; doi:10.1371/journal.pone.0238500)

# SciGenom Trace Viewer

Sample :E4.12\_E4.FP\_26280-3\_8173  
Trim Start :29  
Trim End :203  
Qv20 Bases :174

Run start: 2016/02/23 19:11:33  
Run stop: 2016/02/23 21:23:31  
PDF created: 2016/02/24 09:06:12

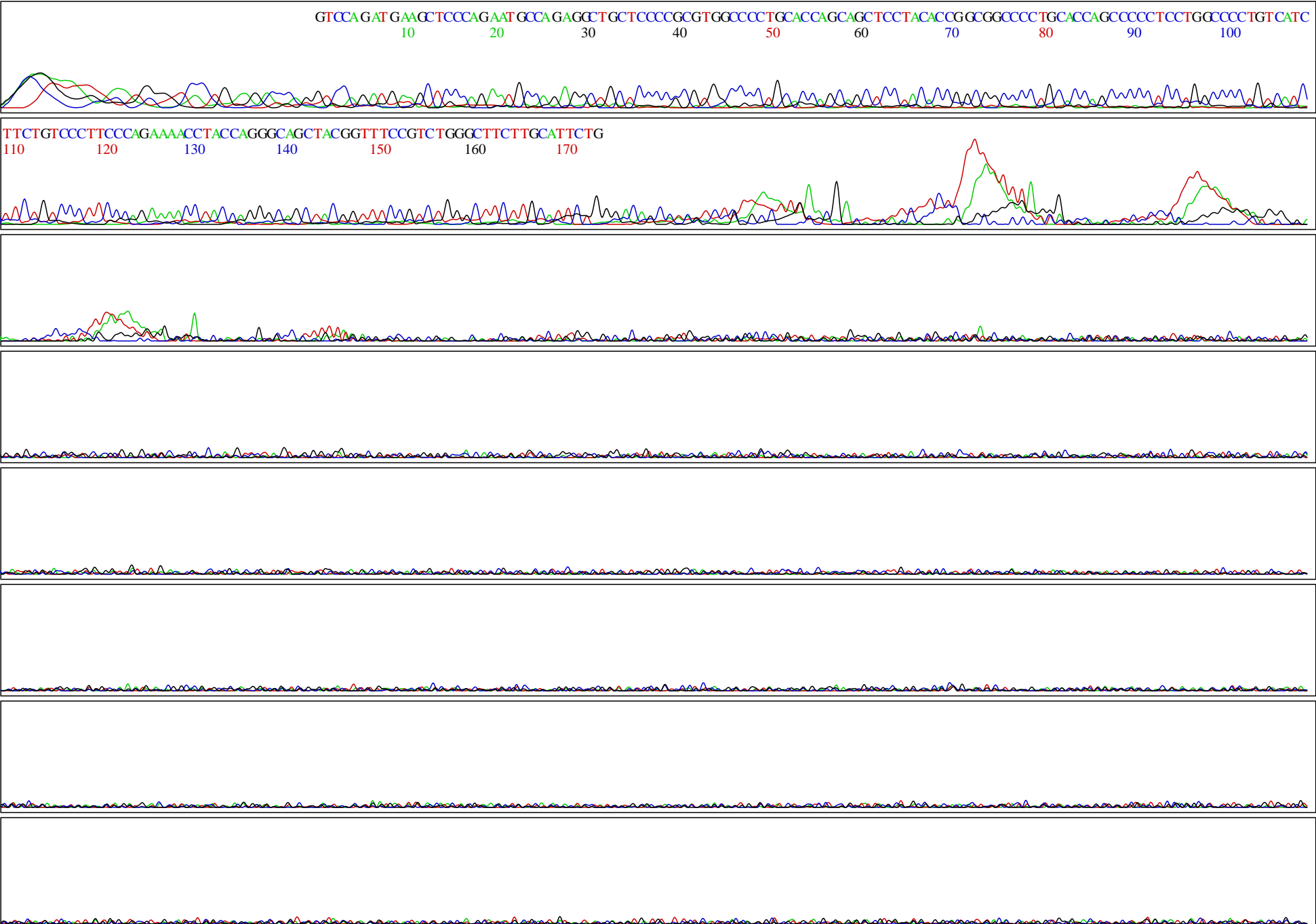

Supplement: S4 File — (PDF) [file pone.0238500.s005.pdf]

# SciGenom Trace Viewer

Sample :E4.13\_E4.FP\_26280-4\_8173  
Trim Start :85  
Trim End :108  
Qv20 Bases :23

Run start: 2016/02/23 19:11:33  
Run stop: 2016/02/23 21:23:31  
PDF created: 2016/02/24 09:06:14

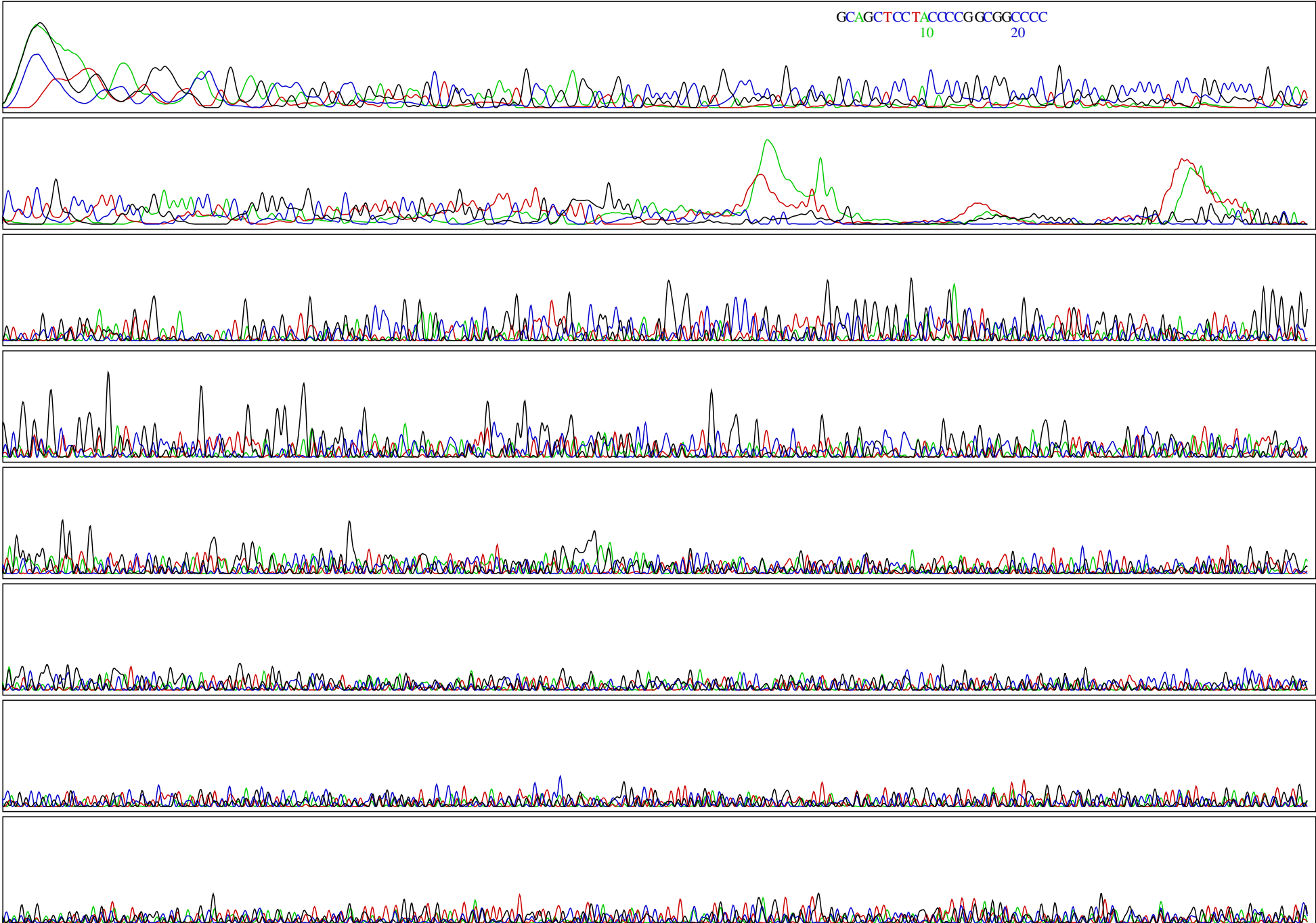

Supplement: S5 File — (PDF) [file pone.0238500.s006.pdf]
