# Supplementary material for: Exploring the p53 connection of cervical cancer pathogenesis involving north-east Indian patients
Source: PLoS One. 2020 Sep 25;15(9):e0238500. doi: 10.1371/journal.pone.0238500 (PMC7518589; doi:10.1371/journal.pone.0238500)
Supplement: Attachment — Submitted filename: Comments.doc [file pone.0238500.s008.doc]

In the manuscript entitled “Screening the p53 Connection of Cervical Cancer Pathogenesis Involving North-East Indian Patients” the authors have discussed the mutation and expression aspects of p53 in cervical cancer pathogenesis

Though the study is relevant as well as conceptually and methodologically sound, however suffers from writing and presentation issues, therefore need major revision.

Some of the major and minor points are as follows:

Abstract:

Background: “Cervical cancer (CaCx) is a global issue” how?? Needs clarity

Human papilloma virus should read: Human papillomavirus

Blood was collected but how was it utilized is not clear??

Keywords: CaCx should be deleted

**Introduction:**

It is a leading cause of cancer related mortality in Indian women of both rural and urban areas??

Well breast cancer in India leads! Latest reference/ GLOBOCAB report should be referred

First Para: last sentence “biomarker” to be deleted

Ref. no. 8 and 9 do not relate to the statements made.

The statement “The data in the role of dysregulated p53 in CaCx pathogenesis has been suggested, but differences exists in the data documented from different geographical niches” needs elaboration and citation.

First page: last sentence: Tp53 should read: TP53

The statement “Genetic alterations in p53 gene have **seldom** been reported in carcinogenesis event [11]” is baffling.

The authors should explain the ration behind this statement or it should be deleted. There is no dearth of reports on the genetic alterations of p53 in cancer.

Last paragraph: Mizoram study??? Cancer of other etiology?? Which cancer?? Reference is missing?? The paragraph is ambiguous

**Materials and methods**

**Enrolment of Patient and sample collection:** Authors have discussed the processing of the samples. The title should be modified accordingly.

Statement on ethical approval is missing.

The ethnicity of the population needs clarity, and how the present population is different from the other population of their country.

Did the patient samples (blood and biopsies) were collected by Gynaecologist or by the non-clinicians under the supervision of Gynaecologist. The statement needs to be reframed.

Why blood was collected??

(for RNA bases study) ??

The following statement should be the part of results and not materials and methods

“and based on the screening report, 84% (N=72) of cervical cancer cases were found to be HPV positive.”

**TP53 expression analysis**

Sybr green should be SYBR Green

IHC: scoring system not mentioned

**TP53 Epigenetic profiling:**

the paired HPV positive cases (i.e. cases with both cancerous and noncancerous

region) specifically for data specificity.

Above statement is not clear: what about HPV negative cancer cases

At many places Kit nomenclature is incomplete, country of origin is missing

Several sites of TP53 known to be acetylated e. g, C-terminal K370, K372, K373, K381, K382, as well as K120, K164, K305 etc. However, rational of selection K305, K373 and K382 should be discussed.

**P53 Acetylation Study**

For which type Ab, WB and IHC was used?? Details should be provided here

**Results**

**Demographical and clinical profile of the enrolled patients**

Clinical profile is missing.

**No association of the results with different stages of CaCx has been made and discussed**

Percentage missing in HPV results

How stage III can be common to both low and high stages of cervical cancer: “lower severity stage (≤stage III) compared to higher stages of CaCx (≥stage III)”

“To check the role of altered p53 gene in the susceptibility to CaCx the mutation study for six

different exons (exon4🡪9) was done by PCR direct sequencing method (Fig 4A)” lacks clarity and should be reframed

“The analysis result from the sequencing showed changes in exon4 of the p53 gene only in the affected region of the studied cases” ??

What is “the affected region of the studied cases”

The following statement should be shifted to the discussion part. “This data suggests that p53 downreglation may probably a crucial early event in the pathogenesis of CaCx pathogenesis in HPV infected cases“

Following statement is the part of results and must be shortened and shifted to the discussion

“Both the two isoforms of p53 due to polymorphism at codon 72 differ in biochemical and

biological properties, especially in reference to its property of inducing apoptosis [19].

Sporadic report also is suggestive of the significance of HPV onco-protein E6 in targeted

degradation of Arg72 p53, thereby altering cellular apoptosis and predisposing individuals to

HPV-related cervical cancers [20]. Secondly, literature suggests that Arg/Pro germline

heterozygotes is retained in squamous cell carcinomas and is more potent in neutralizing p73-

induced apoptosis and also cooperates with other oncogenes to transform cells [21].”

Both the two isoforms of p53??

“The data is therefore suggestive of the prognostic significance of the rs1042522 polymorphism in the studied population, and especially the Arg allele in

HPV+ve cases”

Must go to discussion

When the cases where changes in p53 promoter methylation changes observed were considered ??? Needs clarity

Acetylation also plays a key role in p53 protein stabilization and transcriptional regulations.

Key acetylation sites which is associated with p53 transcript stability which inturn p53

protein expression K305, 373 and 382 was analyzed for differential expression with p53-

acetylation specific antibodies by either western blot analysis (Ac-305) or by immunohistochemistry (Ac-372 and Ac-382).

The above statement cannot be part of the results: half goes to MM and rest to discussion

**Discussion:**

Josefesson A et al. 1998	should read: Josefesson et al. (1998)

There are many such incorrect citations throughout the manuscript

“In Indian females belonging to both urban as well as rural area, cervical cancer has emerged

to be major cause of cancer associated deaths” OR CaBr.

Following statement is too long and lacks clarity

“As per the National Centre for Disease Informatics and Research [24] based report on Cancer Burden in North Eastern States of India, the prevalence of cancer cervix in northeast India stands at 12.3 (10.1) [relative proportion %age (AAR)]; and more importantly, several districts of Arunachal Pradesh, Mizoram, Assam and Nagaland have high to very high distribution of the disease (rate per 100,000) and ranks amongst the highest prevalent areas or districts in India; making it a major health issue amongst females”

“Although infection with high risk HPV strains has been underlined as the driving force in cervical cancer pathogenesis [25]. along with the data from the present studied cohort”

What about high-risk HPV stains’ data in the present cohort. No mention has been made in the present study. Authors should provide data about high-risk HPVs analysed and their association.

If northeast states are Arunachal Pradesh, Mizoram, Assam and Nagaland of India then from which state patients were recruited. Rational for selecting NE patients is not mentioed?? How this population ethinically differs from the other part of the country. Future comparative studies will other ethnic groups will be required to get a clear picture. This needs to be discussed.

“Given the differences in cervical cancer distribution in different geographical

subsets of populations with distinct and different ethnicity, the understanding of these key

molecular and cellular factors becomes relevant and holds clinical significance”

Reference is missing

“Our findings correlate with several studies which demonstrates that in cervical cancer

carcinogenesis the down-regulation of p53 levels is thought to be a key factor [26]”

In the above statement authors says “several studies”, however only one reference has been cited and this reference is not a review article.

What about p53 expression in HPV negative CaCx cases: No indication in results and discussion

As cancer is known to be a multigene effect, how authors can signify the importance of only p53 and not the other genes. This needs to be argued.

Table 1 . p53 should be italicised

Table 2: Number of cases [%age].

% not calculated

Table 2 and 3: CaCx	should read: cervical cancer

At many places figure legends are incomplete., e.g., Fig 4A

What is Con?? Fig A, B,C??

The authors should avoid using “to check” and instead relevant synonyms should be used.

There are innumerable full stops in the text in-between the statements.

e.g., Inactivation of p53 has been reported to be due to hyper-methylation of the promoter region [14]. and it has been associated with human neoplasia [15].

A review of English by a native English speaker or assistance from profession English editing services is strongly recommended.
