# Supplementary material for: Exploring the p53 connection of cervical cancer pathogenesis involving north-east Indian patients
Source: PLoS One. 2020 Sep 25;15(9):e0238500. doi: 10.1371/journal.pone.0238500 (PMC7518589; doi:10.1371/journal.pone.0238500)
Supplement: Attachment — Submitted filename: Response to reviewers.doc [file pone.0238500.s010.doc]

PONE-D-19-32000R1

Exploring the p53 Connection of Cervical Cancer Pathogenesis Involving North-East Indian Patients

PLOS ONE

Dear Dr. Husain,

Thank you for submitting your manuscript to PLOS ONE. After careful consideration, we feel that it has merit but does not fully meet PLOS ONE’s publication criteria as it currently stands. Therefore, we invite you to submit a revised version of the manuscript that addresses the points raised during the review process.

We would appreciate receiving your revised manuscript by May 22 2020 11:59PM. When you are ready to submit your revision, log on to https://www.editorialmanager.com/pone/ and select the 'Submissions Needing Revision' folder to locate your manuscript file.

If you would like to make changes to your financial disclosure, please include your updated statement in your cover letter.

To enhance the reproducibility of your results, we recommend that if applicable you deposit your laboratory protocols in protocols.io, where a protocol can be assigned its own identifier (DOI) such that it can be cited independently in the future. For instructions see: http://journals.plos.org/plosone/s/submission-guidelines#loc-laboratory-protocols

Please include the following items when submitting your revised manuscript:

A rebuttal letter that responds to each point raised by the academic editor and reviewer(s). This letter should be uploaded as separate file and labeled 'Response to Reviewers'.

A marked-up copy of your manuscript that highlights changes made to the original version. This file should be uploaded as separate file and labeled 'Revised Manuscript with Track Changes'.

An unmarked version of your revised paper without tracked changes. This file should be uploaded as separate file and labeled 'Manuscript'.

Please note while forming your response, if your article is accepted, you may have the opportunity to make the peer review history publicly available. The record will include editor decision letters (with reviews) and your responses to reviewer comments. If eligible, we will contact you to opt in or out.

We look forward to receiving your revised manuscript.

Kind regards,

Kalimuthusamy Natarajaseenivasan

Academic Editor

PLOS ONE

**Reviewers' comments:**

**Reviewer's Responses to Questions**

**Comments to the Author**

1. If the authors have adequately addressed your comments raised in a previous round of review and you feel that this manuscript is now acceptable for publication, you may indicate that here to bypass the “Comments to the Author” section, enter your conflict of interest statement in the “Confidential to Editor” section, and submit your "Accept" recommendation.

Reviewer #1: All comments have been addressed

Reviewer #2: All comments have been addressed

2. Is the manuscripts technically sound, and do the data support the conclusions?

The manuscript must describe a technically sound piece of scientific research with data that supports the conclusions. Experiments must have been conducted rigorously, with appropriate controls, replication, and sample sizes. The conclusions must be drawn appropriately based on the data presented.

Reviewer #1: Yes

Reviewer #2: Partly

3. Has the statistical analysis been performed appropriately and rigorously?

Reviewer #1: Yes

Reviewer #2: No

4. Have the authors made all data underlying the findings in their manuscript fully available?

The PLOS Data policy requires authors to make all data underlying the findings described in their manuscript fully available without restriction, with rare exception (please refer to the Data Availability Statement in the manuscript PDF file). The data should be provided as part of the manuscript or its supporting information, or deposited to a public repository. For example, in addition to summary statistics, the data points behind means, medians and variance measures should be available. If there are restrictions on publicly sharing data—e.g. participant privacy or use of data from a third party—those must be specified.

Reviewer #1: Yes

Reviewer #2: No

5. Is the manuscript presented in an intelligible fashion and written in standard English?

PLOS ONE does not copyedit accepted manuscripts, so the language in submitted articles must be clear, correct, and unambiguous. Any typographical or grammatical errors should be corrected at revision, so please note any specific errors here.

Reviewer #1: Yes

Reviewer #2: Yes

**6. Review Comments to the Author**

**Reviewer #1 Comment**:

In the manuscript entitled “Exploring the p53 Connection of Cervical Cancer Pathogenesis Involving North-East Indian Patients” the authors have discussed the mutation and expression aspects of p53 in cervical cancer pathogenesis

The authors have satisfactory modified the manuscript. However, few minor concerns need to be addressed before final acceptance.

**Materials and methods:**

- In: HPV screening and genotyping by PCR

“using the specific primers for HPV16 1nd HPV” ???..Please clarify… “1nd”

**Author Response:** **It was typographical error. Instead of “1nd” it should have been “and” and the necessary correction has been made in the text and the same has been highlighted in blue. We apologize for the mistake**

- I am failing to understand that, when authors have performed HPV16 and 18 detection using type specific primers, then why the results pertaining to HPV16 and 18 frequencies have not been incorporated the results section. If at all cases were HPV16 positive than it should be highlighted in the results

**Author Response: The table has been modified as per the Reviewer’s kind suggestion. As a part of an initial screening process we performed the genotype analysis for both HPV16 and HPV18 and the data showed the presence of HPV16 genotype only in the studied cohort. The line stating the same was previously missing but is now included in the text. The modified table including the details of HPV genotype data has also been included, and the same may kindly be considered at your end.**

- Moreover, the screening results of HIV, Hepatitis’s virus etc in the blood samples should be also highlighted. Further, the authors should clarify “etc” pathogens.

A brief mention of methods of detection of “HIV, Hepatitis’s virus etc” should be made.

**Author Response: As suggested we have provided the brief description for detection method for pathogenic screening, as well the screening results in the materials and methods and result section of the manuscript.**

- Again the spelling of “Josefesson” is wrong in the text. It should read “Josefsson”.

Authors should rigorously check the spelling of all the citations made in the text and reference list.

**Author Response: We apologize for the mistake. As suggested the necessary correction has been made in the manuscript and the same has been highlighted in blue.**

**Reviewer #2: Review Comments**

**Response to query 2**

I feel that the results which are not statistically significant should also be mentioned with p-values in the manuscript or else the information seems incomplete.

**Author response: As suggested, we have incorporated the p-values for the statistically insignificant result as well and the same have been highlighted in blue in text.**

**Response to query 3**

As mentioned by the author in the manuscript “Further the blood samples were used to screen the presence of any other pathogenic infection such as HIV, Hepatitis’s virus etc apart from HPV in the collected samples.” As per the above statement I would like to know whether HPV was tested from the blood samples. If so, please mention how one could assess the presence of HPV infection from blood samples.

**Author response: No, the presence of HPV infection was not assessed from blood samples, it was determined from the cervical tissues. The blood samples were used to rule out the presence of any other pathogenic infections (as mentioned in the results section) as the exclusion criteria of our study.**

**Response to query 6**

Please include all the results with their statistical value in a single table format for better visualization and understanding.

**Author response:**

**The difference in the relevant p53 data at all levels and their statistical differences have been either illustrated in the figures 1, 2A, 2B and 4C as well as in Table 3, or have been encrypted in the text under different results sections. So we feel that compiling the data set under one common table will be a duplication of data representation, and also make the data set quite complex (as different parameters results have been discussed under different sub-sections under the results. But based on the suggestion, we have now tabulated the p53 exon 4 genotyping data as supplementary table for ready reference and consideration at your end.**

**Supplementary Table: Details of p53 exon4 polymorphism based difference in genotype analysis**

| **Stages** | **HPV +ve (N=72)** | | | | | **HPV -ve (N=13)** | | | | |
| --- | --- | --- | --- | --- | --- | --- | --- | --- | --- | --- |
|  | N | Pro/Pro | Arg/Pro or Arg/Arg | P value | ODDs ratio | N | Pro/Pro | Arg/Pro or Arg/Arg | P value | ODDs ratio |
| **IIA and IIB** | 48 | 9 [18.75] | 39 [81.25] | ref | 0.877 (0.258-2.979) | 09 | 5 [55.55] | 4 [44.45] | ref | 1.250 (0.118- 13.240 |
| **IIIA, IIIB**  **and IV** | 24 | 5 [20.83] | 19 [79.17] | 0.834 |  | 04 | 2 [50.00] | 2 [50.00] | 0.859 |  |

**Response to query 8**

As mentioned by the author the antibody used for immunohistochemistry was ab26 (abcam) however in the datasheet of the company it is mentioned that this antibody was not tested for IHC by the company. So, if you could kindly clarify and justify why this antibody was chosen for immunohistochemistry?

Author has mentioned in the response 8 that “The antibody used i.e. ab26 (Abcam, UK) is anti-p53 monoclonal antibody capable of recognizing both mutant forms and wild-type human p53.” So I would like to know how you can differentiate which expression is due to mutant form and which is due to wild type human p53.

**Author response: In total two anti-p53 antibodies were used for p53 expression studies based on IHC. The first antibody was specifically for IHC, but didn’t yield the expected acceptable results. The second anti-p53 antibody (ab26, Abcam) was chosen for its capabilities to recognize both mutant and wild type conformational forms of p53. Although Abcam (Abpromise guarantee) states that it covers the use of ab26 in IP, ICC/IF, WB; but didn’t specified that it cannot be used for other applications like IHC. We therefore experimentally performed and validated the results with the ab26 p53 antibody by IHC under the supervision of a senior pathologist. Henceforth, the utility of the ab26 p53 antibody for IHC was also demonstrated experimentally. Unfortunately, one cannot claim directly that the p53 protein expression was based on which conformational form of p53 (as ab26 detects both the conformations), but it could be done in association with the genotype data. But, as the post-transcriptional modifications and regulations of p53 are now been realized as an important aspect and complex (outside the purview of the present work), therefore we have not commented on the association of p53 protein expression with the p53 genotype data (only the association of p53 mRNA data with the p53 genotype data was analyzed and documented).**
